# Multicolor 4D Fluorescence Microscopy using Ultrathin Bessel Light Sheets

**DOI:** 10.1038/srep26159

**Published:** 2016-05-18

**Authors:** Teng Zhao, Sze Cheung Lau, Ying Wang, Yumian Su, Hao Wang, Aifang Cheng, Karl Herrup, Nancy Y. Ip, Shengwang Du, M. M. T. Loy

**Affiliations:** 1 Department of Physics, The Hong Kong University of Science and Technology, Clear Water Bay, Kowloon, Hong Kong, China; 2 College of Life Sciences, South China Agricultural University, Guangzhou 510642, China; 3 Division of Life Science, The Hong Kong University of Science and Technology, Clear Water Bay, Kowloon, Hong Kong, China; 4 State Key Laboratory of Molecular Neuroscience, The Hong Kong University of Science and Technology, Clear Water Bay, Kowloon, Hong Kong, China; 5 Division of Biomedical Engineering, The Hong Kong University of Science and Technology, Clear Water Bay, Kowloon, Hong Kong, China

## Abstract

We demonstrate a simple and efficient method for producing ultrathin Bessel (‘non-diffracting’) light sheets of any color using a line-shaped beam and an annulus filter. With this robust and cost-effective technology, we obtained two-color, 3D images of biological samples with lateral/axial resolution of 250 nm/400 nm, and high-speed, 4D volume imaging of 20 μm sized live sample at 1 Hz temporal resolution.

The twin goals of high spatial and high temporal resolutions in live imaging of biological samples have been elusive until the recent progress in light-sheet microscopy^1^,^2^,^3^,^4^,^5^,^6^, especially the ground-breaking invention of the lattice light-sheet microscope (LLM)^7^ in which ‘non-diffracting’ ultrathin light sheets are employed. The unparalleled capability to perform live imaging of objects ranging in size from molecules to embryos with high spatiotemporal resolution and minimal phototoxicity, has attracted much attention. The crafting of an ultrathin lattice light-sheet (LL) starts from writing a 2D optical lattice pattern onto a spatial light modulator (SLM). Incident light diffracted by this SLM is then filtered by an annular ring mask (“annulus”) to give the ‘non-diffracting’ Bessel property. The optical lattice pattern is then dithered to form a desired lattice light sheet. For each excitation light frequency, a different pattern is written onto the SLM, and for multicolor imaging, the lattice pattern must be rapidly switched and synchronized to the color changes. Despite the many advantages of this method, the efficiency in producing the LL is very low: less than 1% of the light gets to the sample, and the source of this inefficiency can be traced to the fast-switching SLM.

Here we demonstrate a technique, Line Bessel Sheet (LBS) (Fig. 1), to produce ultrathin ‘non-diffracting’ Bessel sheets without the cost, complexity and inefficiency of the SLM. The excitation light is made line-shaped (e.g. by passing through a slit, or other means, see Supplementary Note 1) and then passes through an annulus imaged to the back focus plane of the excitation objective (Special Optics, numerical aperture (N.A.) 0.7). The line image on the sample will be in focus for an extended length^8^,^9^ along the propagation direction, forming a ‘non-diffracting’ Bessel sheet (Fig. 1a, Supplementary Note 1)^10^,^11^,^12^. We demonstrate that it is possible to design a combination of line thickness and annulus size so that input laser light of *any* color will emerge as ultrathin light sheet of that color (Supplementary Movie 1) without dithering and without switching any components or mask patterns. As the excitation ultrathin light sheet optical setup is totally passive, no complex synchronization is required other than the change of laser color with the camera frame for sequential multicolor imaging. Simultaneous multicolor imaging is also feasible.

With the exception of the illumination arm, the main body of our microscope is constructed following the design of the LLM^7^ with some modifications (Supplementary Figure 1a). We designed two line thickness-annulus combinations: LBS1 and LBS2, each with different characteristics (Supplementary Note 1). In LBS1 (Fig. 1b), the central light sheet thickness is matched to the axial resolution of the detection objective (600 nm), maximizing the detection efficiency and minimizing photo-bleaching. This ultrathin light sheet maintains its shape for over 15 μm, compared to 3 μm for a Gaussian light sheet of the same 600 nm thickness (Fig. 1f). In LBS2 (Fig. 1c), we crafted a thinner central sheet (~400 nm), which results in higher intensity side lobes. With proper deconvolution, however, the side lopes can be removed, yielding an overall deconvoluted point spread function (PSF) of ~400 nm (Supplementary Figure 2a,b). This higher resolution ultrathin light sheet can maintain its shape for over 10 μm, compared to about 1 μm for a Gaussian light sheet of the same thickness. The LBS1 and LBS2 schemes were experimentally tested and validated using 20 nm beads tagged with the fluorescent proteins, GFP and mCherry, excited with light at 488 nm and 560 nm respectively (Fig. 1d,e). These results are in excellent agreement with theory (Fig. 1b,c).

We demonstrate the capability of LBS by acquiring 3D two-color images of microtubules and mitochondria in fixed cells from cultures of the HT22 neuroblastoma cell line. The size of the sample was 50 μm in x, 35 μm in y and 20 μm in z. As shown in Supplementary Figure 1a, the illumination arm was completely fixed and passive from the fiber exit to the sample. The detection arm was also fixed. The sample was scanned using a piezo stage, moving at a direction 30^o^ to the ultrathin sheet and 60^o^ to the detection objective (Fig. 2a). At each stage position, the camera sequentially captured the fluorescence from the two colors as shown in Supplementary Figure 2b. As expected, single slice images showed sharply defined axial resolution and high signal-to-noise ratio (SNR) over the length of the light sheet (~10 μm) for both LBS1 and LBS2, in sharp contrast to that of the Gaussian sheet (i.e., without the annulus) which remained thin for only ~3 μm. This resulted in seriously deteriorating SNR outside of this short region (Fig. 2b). The lateral resolution using the high NA detector objective (Nikon, N.A. = 1.1) was about 250 nm. The axial resolution was determined by the thickness of the sheet, which is ~600 nm for LBS1 and ~400 nm for LBS2 (with deconvolution) (Supplementary Figure 3). Significantly, the resulting high resolution two-color images can be obtained quickly and at very low laser power. The fluorescence image of each ultrathin slice can be captured in wide field within 10 ms, using excitation laser power as low as 25 μW. With both excitation and detection arms stationary, volume imaging can be achieved by scanning the sample to produce a stack of two-color slices. These 3D stacks can be easily ‘de-skewed’ to form two-color volume images (Fig. 2c,d). As these volume images are composed of stacks of 500 two-color slices separated by 100 nm the capture time volume for the entire volume is ~10 sec. The resulting images show crisp and well resolved representations of the cellular system of microtubules (green) and mitochondria (red) typically found in these cells.

LBS1 and LBS2 both can image with low photo-bleaching (See Supplementary Figure 4). While LBS2 (with deconvolution) yields better axial resolution (Supplementary Figure 3d), LBS1 excels in minimizing photo-bleaching: with its higher proportion of excitation power in the central peak, less total power is required to get the same SNR.

LBS techniques are particularly well suited to obtain multicolor 4D movies for dynamic study of live cells given its excellent spatial resolution, low photo-bleaching, and fast volume capture rate. To demonstrate this, we performed two-color live imaging of tobacco BY-2 cells where endocytic vesicles were marked by a GFP fusion protein (GFP excitation at 488 nm) and the plasma membrane was labelled by FM 4-64 (excitation at 560 nm), a well characterized fluorescent dye used to trace cell endocytosis^13^. With LBS1, we scanned a 20 μm sized volume containing 50 two-color slices at 1Hz (10 ms per slice per color) such that the rapid endocytosis process^13^,^14^ could be resolved temporally with no lagging between colors (Fig. 2e and Supplementary Movie 2). The spatial resolution (250 nm lateral and 600 nm axial) was high enough to clearly visualize the 3D distribution of the vesicle at any time point. Because only 20 μW laser power reached the sample, LBS1 resulted in ultra-low photo-bleaching that allowed for imaging over 15 mins at 100 Hz (1000 time points of whole volume imaging) with no deterioration of the quality of the two fluorescent markers (Supplementary Figure 4c).

In conclusion, LBS microscopy builds on the ground-breaking work that established light-sheet illumination as a new and powerful optical imaging method of live biological samples. LBS2 has excellent lateral and axial resolution (250 nm/400 nm); and LBS1 has good resolution (250 nm/600 nm axial/lateral) and very low level of phototoxicity that allow capture of over 1000 volumes or 100,000 slices in live plant cells without obvious reduction in SNR or altering the physiology state of the specimen, at a frame rate to yield 1 Hz time resolution over 20 μm scanning range. LBS does not use a SLM and is very efficient: up to 50% of the laser light gets to the sample, many fold improvement over the LL. With this high efficiency, inexpensive low power lasers can be used. LBS can operate at any color without switching elements, and is capable of simultaneous multicolor ultrathin light-sheet imaging. With passive and stationery excitation and detection arms, the system is robust and easy to operate. Its simplicity of design and much improved efficiency in producing the ultrathin light sheets means that the advantages of light-sheet imaging can now be made accessible to many more researchers.

## Methods

### System Setups

Supplementary Figure 1a shows the structure of a LBS microscope. Lasers (MPB communications) at 488 nm and 560 nm modulated by an AOTF (Crystal technology) are delivered through a single mode fiber (not shown in figure). Fiber output beam is collimated by an f = 50 mm lens then shaped by a pair of cylindrical lenses (f = 50 mm and f = 300 mm) such that it passes through the 200 μm wide single slit with 85% efficiency, forming the diffraction pattern onto the annulus after the Fourier transform lens with f = 500 mm. Detailed design information regarding LBS1 and LBS2 is available in Supplementary Note 1. A telescope expands the cropped diffraction pattern by 2.5 times and projects it to the back pupil plane of the excitation objective (Special Optics 28.6×, NA = 0.65 WD = 3.5 mm), which will perform Fourier transform and produce LBS at its sample plane. The focal plane of the detection objective (Nikon Apo LWD 25×, NA = 1.1 WD = 3 mm; mounted perpendicular to the axis of excitation objective) is then carefully aligned to LBS axis so the overall point spread function appears symmetric. With the relative position between the excitation and emission objectives fixed, the robust alignment between objectives can be maintained for months without the need for realignment. Specimens are mounted on a 5mm diameter round coverslip held by a piezo stage (Physik Instrumente) able to translate along the plane of coverslip. Coverslip is set at a 30° angle to the axis of the excitation objective and 60° angle to that of detection objective. The tips of two objectives and the coverslip are all immersed in PBS or medium for live cell imaging. The fluorescent signal from the sample is collected by the detection objective and focused on to sCMOS camera (Hamamatsu) by a tube lens with f = 600 mm. A multiband emission filter designed for GFP and mCherry (Semrock) is placed in front of the tube lens to block unwanted illumination from the excitation laser. The major structures of the microscope including mounting of objectives, sample holders and perfusion bath are directly adopted from the designs in LLS.

For volume imaging, the sample driven by the piezo stage is scanned by the fixed LBS over the entire region of interest (also known as the sample scan mode). In operation, as illustrated by Supplementary Figure 1b, the camera is triggered at a frame rate subject to the requirement of the imaging, normally 100 Hz, in synchronized read-out mode. The camera outputs the timing of global exposure, which is used to synchronize AOTF as well as the piezo stage, so that the laser illuminates the sample only during the global exposure period, and the piezo moves a step forward during the period when laser is turned off. In dual-color operation, laser wavelengths are switched at every frame, and the piezo stage steps up after both colors are imaged sequentially. We also note that multicolor LBS ranges from 400 mm to 1200 nm can be generated simultaneously using the same slit and annulus. Simultaneous multicolor imaging seems feasible by incorporating a channel splitter^15^.

### Image Processing

The raw image stacks acquired via sample scan are sheared because the motion of sample is at 60^o^ to the axis of detection objective (Fig. 2a). A Matlab code is applied on entire raw stack to remove such shearing via coordination transformation. The corrected 3D stack is then visualized by ImageJ plugins through maximum projection or 3D rendering.

Lucy-Richardson deconvolution is applied to 3D image stacks captured by LBS2 using PSF model obtained by nonspecifically bounded dye molecules or florescent beads coated on coverslips. This approach aids the visualization by restoring the regular shape of the axial point spread function.

### Sample Preparation and Imaging Conditions

**Figure 1:**
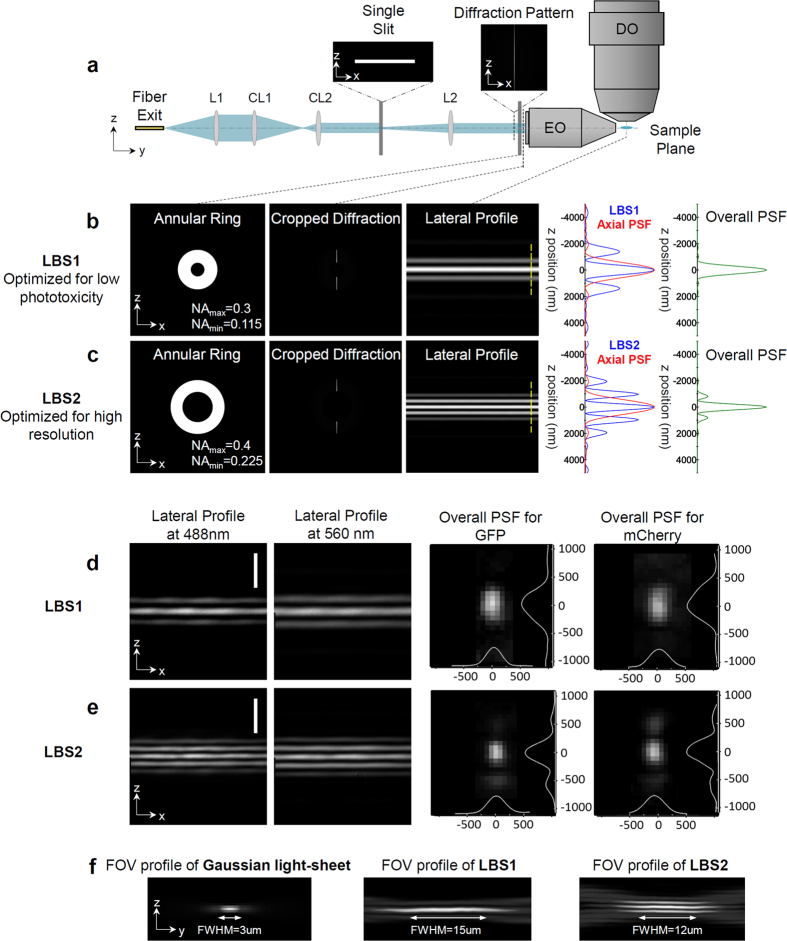
Generation of LBS with a single slit and an annulus. (**a**) A simplified schematics illustrating LBS generation. L1: Collimating lens; CL1 and CL2: a pair of cylindrical lenses shaping the beam appropriately to maximize the energy passing through the single slit; L2: Fourier transform lens; EO: Excitation objective; DO: Detection objective. The beam is defined as propagating along y-axis. Using custom designed annuli one can craft LBS optimized for (**b**) ultralow phototoxicity (LBS1) or (**c**) high resolution (LBS2). In (**b**,**c**) from left to right: the annulus used and its numerical apertures (NA): NA_max_ and NA_min_; the diffraction pattern after the annulus; the cross-section of the LBS generated at sample plane; the intensity plot (blue) along the dotted yellow line and the axial point spread function of the NA = 1.1 detection objective (red); and the axial plot of overall point spread function. (**d**,**e**) show the experimentally generated LBSs at different wavelengths (488 nm for GFP and 560 nm for mCherry). The point spread functions were measured with 20 nm fluorescent beads [left two columns in (**d**,**e**)], which are in good agreement with the theoretical plot in (**b**,**c**). (**f**) The measured FOV profile of Gaussian light-sheet, LBS1 and LBS2. The Gaussian light-sheet is generated by taking away the annulus and confining the maximum NA to 0.5 so that the beam waist has a thickness of 600 nm, which is comparable to the thickness of LBS1. Scale bars in (**d**,**e**): 5 μm.

**Figure 2:**
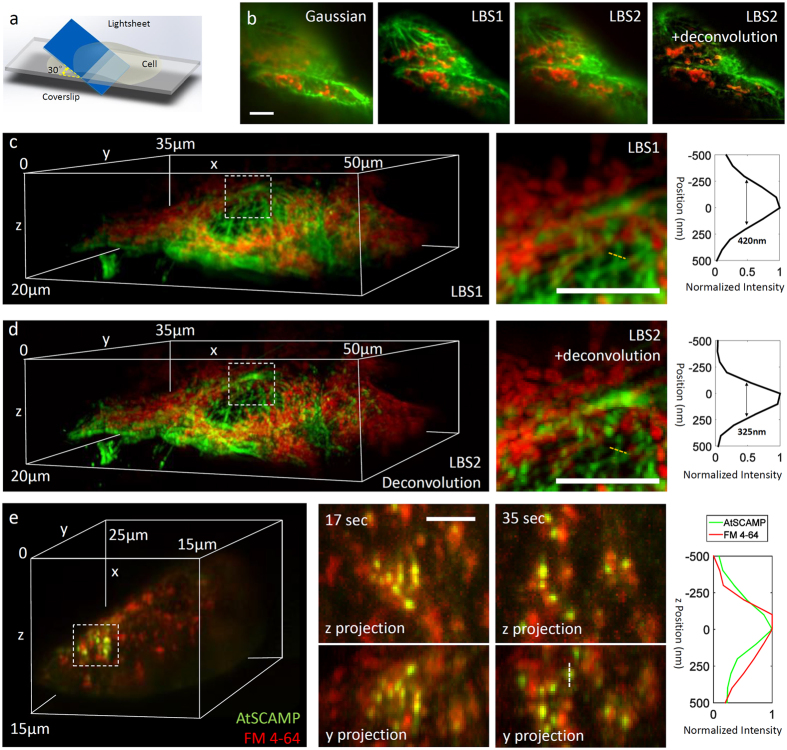
LBS 4D multicolor imaging. (**a**) The illumination geometry of LBS, where the light sheet is tilted 30° to the surface of coverslip. Scanning is done by horizontal movement of the coverslip while keeping the light sheet fixed. Fixed HT22 cells double-stained with Alexa488-tagged anti-tubulin (Green) and Alexa555-tagged anti-Tom20 (Red, and antigen in the mitochondria outer membrane) were imaged by different light sheet configurations in the same region. (**b**) Single slices in a “raw” 3D stack (before de-skewing). In the image obtained by Gaussian light-sheet, the SNR reduced rapidly from the bottom (where the waist of Gaussian light-sheet is aligned) to the top due to the rapid beam diffraction. On the other hand, LBS1 produced optical sections with minimal background and high SNR across the entire field of view. LBS2 exhibits multiple peaks in the axial point spread function, but can be sharpened after deconvolution. (**c**,**d**) show the two-color 3D rendering of HT22 cells (tubulin in green and Tom20 in red) scanned with LBS1 and LBS2 with deconvolution, respectively. From left to right: the two color 3D rendering of the cell; a zoom-in of the boxed region; the intensity plot of the tubulin signal along the doted yellow line. (**e**) 4D multicolor imaging of live tobacco BY-2 cells using LBS1. Green puncta represent the AtSCAMP3-GFP labeled clathrin coated endocytic vesicles, while the red fluorescent FM4-64 dye labels all the endocytic vesicles derived from plasma membrane. The 3D distributions of both types of vesicles are clearly resolved in time and space. From left to right: the 3D rendering of the first time point; the z projection and y projection at the boxed region at different time (the 17 sec and 35 sec), showing the change of vesicle distribution; and the axial profile along a vesicle marked by dotted line, showing the co-localization of two proteins. Scale bars: 5 μm.

Fluorescent beads samples are prepared for PSF measurement (Fig. 1). After cleaning with ethanol, coverslips are coated with a drop of poly-L-lysine for 5 min then washed with water. Fluorescent beads in two colors (20 nm diameter beads mix coated by Alexa 488 and Alexa 555, ThermoFisher) are diluted 1:20,000 upon use. A 50 μl aliquot of the bead suspension is applied to each coverslip for 5 min before washing with double distilled water to remove any unattached beads.

HT22 cells were cultured on the 5 mm coverslips for two days then fixed with 4% paraformaldehyde for 15 min at room temperature. Before immunolabeling, cells were washed three times with PBS. The fixed HT22 cells were then incubated with blocking buffer (5% Donkey Serum with 0.3% Tritox-100 in PBS) for 1 h at room temperature. Cells were then incubated at 4 °C, overnight with mouse anti-β-tubulin (Cell Signaling) and rabbit anti-Tom20 (Santa Cruz) primary antibodies diluted in incubation buffer at 1:500 and 1:200, respectively. The next day cells were washed with PBS three times for 5 minutes each. Secondary antibodies were applied – Alexa488 donkey-anti mouse and Alexa555 donkey-anti rabbit at a dilution of 1:500 for 1 h at room temperature. The coverslips were then rinsed with PBS five times (5 min each).

Live cell imaging was performed using the transgenic tobacco BY2 cell line. For Agrobacterium-mediated transformation, a GFP-AtSCAMP3 plasmid was introduced into Agrobacterium (strain LBA4404) by electroporation before they were used to transfect wildtype tobacco BY-2 cells as described previously^16^. BY-2 cells were maintained in Murashige and Skoog (MS) medium by subculturing twice per week at room temperature in a shaker set at 125 rpm. Transfected BY-2 cells were transferred onto MS medium (Sigma-Aldrich) containing kanamycin (50 mg/mL) and cefotaxin (300 mg/mL) and incubated at room temperature for 3 to 4 weeks until transformed colonies were visible. Resistant cell colonies (about 50 to 100 from each construct) were subjected to preliminary screening for GFP signals and patterns via confocal immunofluorescence. Selected transgenic cell lines (5 to 10 per construct) were further transferred into MS liquid medium containing kanamycin to initiate suspension culture and used for subsequent process. For imaging, 10 μl of cell suspension was placed on a poly-L-lysine coated coverslip and allowed settle for 3 min before flushing away any unattached cells. 10 μL of FM 4-64 solution at 1:3000 dilution was applied directly to the coverslip few minutes before the imaging procedure.

## Additional Information

**How to cite this article**: Zhao, T. *et al.* Multicolor 4D Fluorescence Microscopy using Ultrathin Bessel Light Sheets. *Sci. Rep.* **6**, 26159; doi: 10.1038/srep26159 (2016).

## Supplementary Material

Supplementary Information

Supplementary Movie 1

Supplementary Movie 2
