# Supplementary material for: Multicolor 4D Fluorescence Microscopy using Ultrathin Bessel Light Sheets
Source: Sci Rep. 2016 May 18;6:26159. doi: 10.1038/srep26159 (PMC4870613; doi:10.1038/srep26159)
Supplement: Supplementary Information [file srep26159-s1.pdf]

SUPPLEMENTARY MATERIAL

Multicolor 4D Fluorescence Microscopy using Ultrathin Bessel Light sheets

Teng Zhao, Sze Cheung Lau, Ying Wang, Yumian Su, Hao Wang, Aifang Cheng, Karl Herrup, Nancy Y. Ip, Shengwang Du & M. M. T. Loy

**Supplementary Note 1**	LBS1 and LBS2 designs
**Supplementary Figure 1**	System setups and control sequence
**Supplementary Figure 2**	Deconvolution in LBS2
**Supplementary Figure 3**	Comparing the axial resolutions of LBS1, LBS2 and Gaussian light-sheet
**Supplementary Figure 4**	Photo-bleaching of LBSs
**Supplementary Video 1**	LBS generation at different wavelengths with a fixed set of slit and annulus
**Supplementary Video 2**	4D multicolor imaging of endocytosis Tobacco BY-2 cells

Supplementary Note 1: LBS1 and LBS2 designs

The input line-shape light can in principle be formed using a cylindrical lens or prism pair compressor without a slit, particularly for single color imaging. For multicolour application, however, the slit has the essential role to define the position of the line, independent of color. For this reason, a 200 micron width slit is used throughout this paper.

LBS1 is designed to minimize photo-bleaching by reducing the number and intensities of side-bands, thus maximizing the portion of energy in the center peak, which can be achieved by increasing the width of the annulus, i.e., (NA_{max} – NA_{min}). On the other hand, longer light sheet is obtained with smaller NA_{max}, albeit at the expense of a thicker sheet [1]. With this in mind, for LBS1 we used annulus with NA_{max}=0.3 and NA_{min}=0.112, as shown in **Figure 1b**. The resulting LBS was 15 µm long and having only two weak side-bands, which can be suppressed by the axial detection point spread function, yielding an overall single band PSF of 600 nm (FWHM) axial resolution with exceptionally low phototoxicity (See **Supplementary Figure 4** for more detail.) This 15µm long LBS is sufficient for most cell imaging applications; if needed longer LBS can be crafted by scaling the annulus to smaller dimensions. It is also possible to use phase elements to create multi-foci thus double or triple the LBS length without any change in the cross-section of LBS.

LBS2 aims to achieve higher axial resolution, using an annulus with NA_{max}=0.4 and NA_{min}=0.225, as shown in **Figure 1c**. The light sheet can propagate ~12 µm with a 400 nm thick central band, but flanked by multiple progressively weaker bands. The overall axial PSF consists of this ultrathin central band, plus two weaker side bands which can be removed by deconvolution (**Supplementary Figure 2**.)

While the light sheet thickness is determined by the annulus, the width of the light-sheet (the dimension of LBS in x) is in proportion to the length of the single slit. The18mm long slit length used resulted in a 40µm wide light sheet that is sufficient for most cases.

We found that a slit width of 200 µm appear to be optimal for our particular setup allowing over 50% of exit-fiber laser power to reach the sample for LBS1, or 38% for LBS2 at 488 nm (47% and 35% at 560nm). Multicolor imaging can be done with a fixed set of slit and annulus.

We note that, non-diffracting patterns in any shape can be mathematically expressed in circular coordinates[2,3] (i.e. Bessel beams) or elliptical coordinates (i.e. Mathieu beams[4]), and it is possible to extend the depth of any pattern by producing angular spectrum in the *k*-space with an annular mask[5]. LBS is one of the examples to produce extend light sheet with a pattern (a slit in this case) and an annulus and is thus given the name line Bessel beam.

Reference:

[1] Liang Gao, Lin Shao, Bi-Chang Chen & Eric Betzig, *Nature Protocols* **9**, 1083–1101 (2014).
[2] Z. Bouchal, *Opt. Lett.* **27**(16) (2002)

[3] J. Durnin, *J. Opt. Soc. Am. A*, 4, 651 (1987).
[4] J. C. Guiterrez-Vega et al. *Opt. Commun.*, 195 35-40 (2001)
[5] W. T. Welford, *J. Opt. Soc. Am.*, **50**(8), 749-753 (1960);

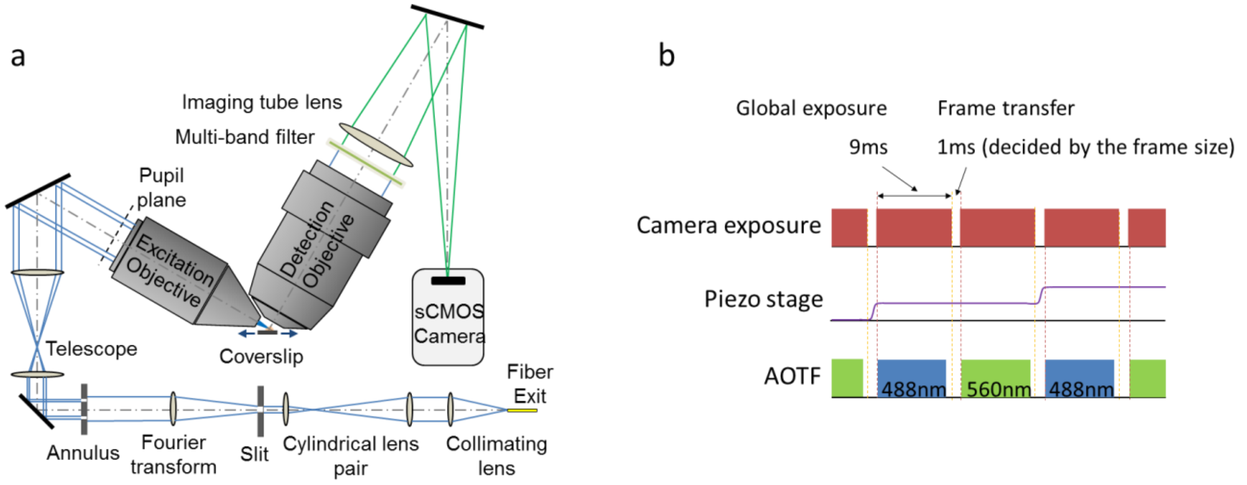

Supplementary Figure 1 | System setups and control sequence. (**a**) System schematics of a LBS microscope. The fiber output was collimated and shaped by a pair of cylindrical lenses to maximize output through a 200 µm slit. An f=500 mm lens is used to perform Fourier transform after the single silt, and project the diffraction pattern onto an annulus. The image of the annulus is zoomed by 2.5 times via a telescope system before projected onto the back focal plane of excitation objective. The LBS is produced at sample plane 30° to the plane of coverslip, and right in the focal plane of the detection objective. With the relative position between the emission and detection objectives fixed, the coverslip is driven by a piezo stage, allowing specimen to be scanned step by step. The fluorescent emission is collected by the detection objective and captured by a sCMOS camera after the multi-band emission filter. (**b**) The triggering sequence used in LBS imaging. Camera running in synchronous readout mode generates high output during the global exposure and low during data transfer. Laser controlled by an AOTF is turned on only during global exposure. For multicolor imaging, the wavelength is switched for adjacent frames. The piezo stage triggered by the falling of global exposure moves a step forward after both colors are imaged.

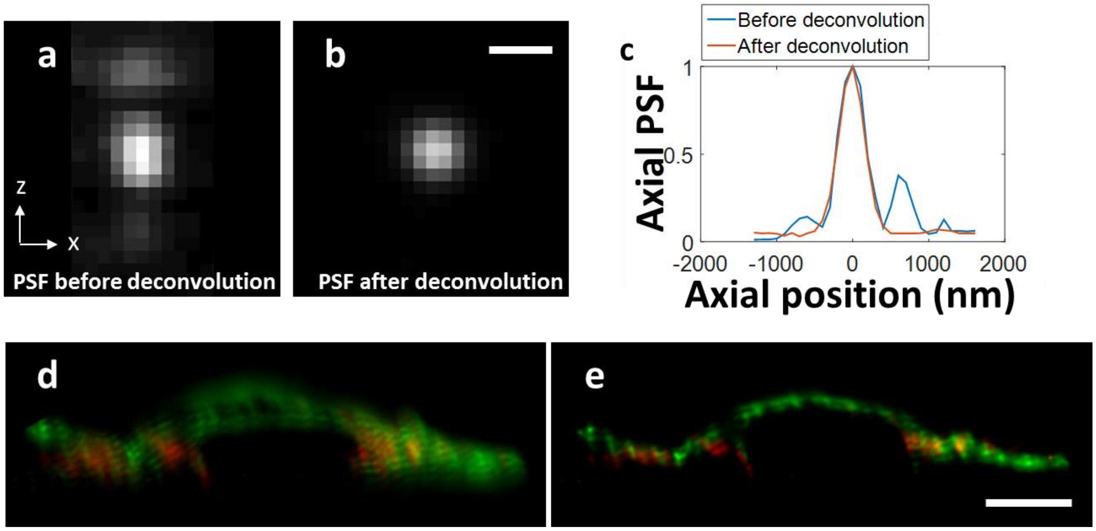

Supplementary Figure 2 | Deconvolution in LBS2. PSF of LBS2 has multiple peaks, which can be removed using deconvolution. (**a**) An image of a dye molecule scanned with LBS2 showing the shape with multi peaks in axial PSF. (**b**) After performing Lucy-Richardson deconvolution to the entire 3D stack, the side peaks in the PSF can be removed. (**c**) The axial plot of the PSFs before and after deconvolution. (**d**) A non-deconvoluted cross-section of a fixed HT22 cell stained with Alexa488 anti-tubulin (Green) and Alexa560 anti-Tom20 (mitochondria outer membrane, Red) imaged by LBS2. (**e**) The same region of (**d**) after deconvolution. Scale bars: 500 nm in **a** and **b**; 5 µm in **d** and **e**.

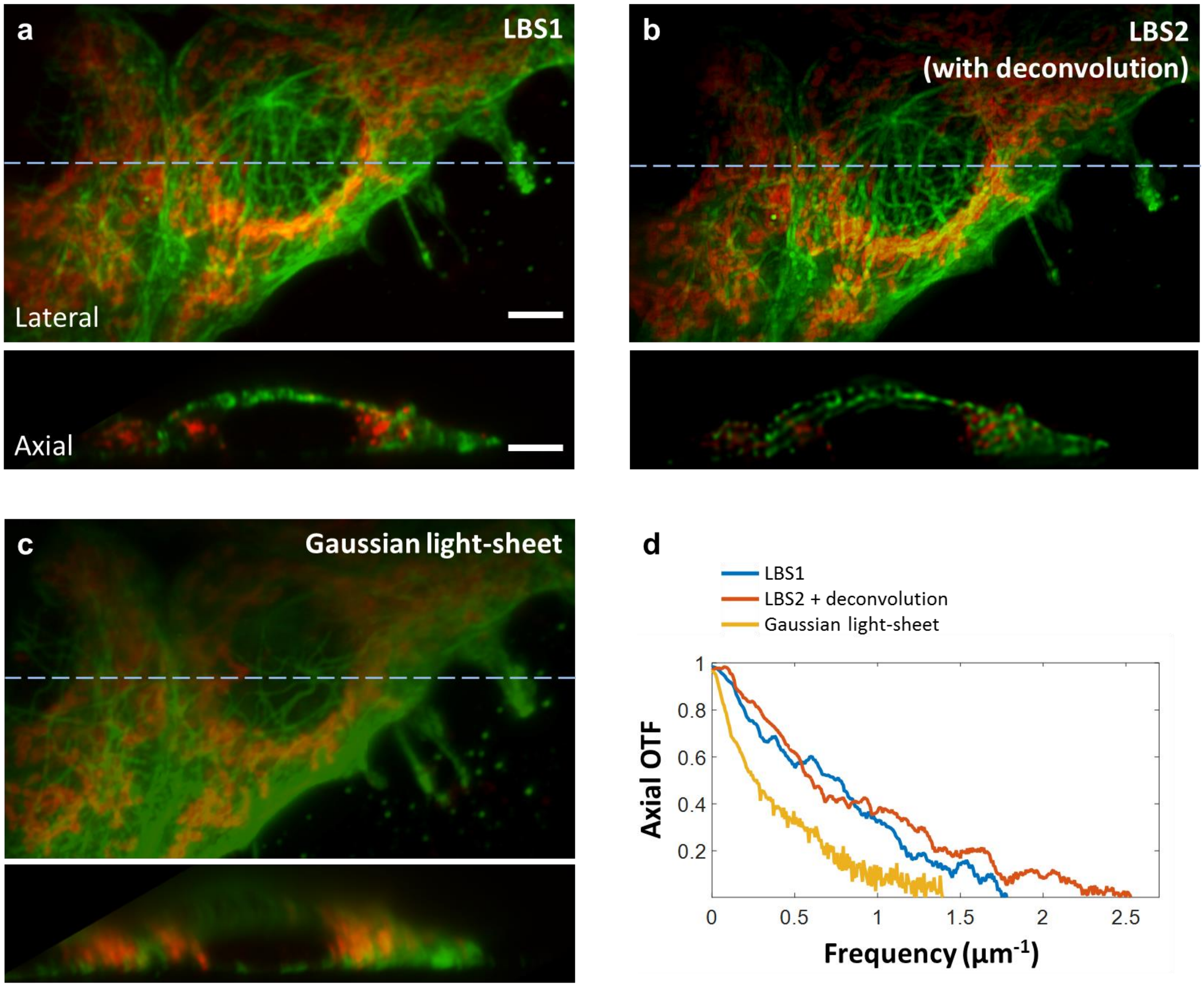

Supplementary Figure 3 | Comparing the axial resolution of LBS1, LBS2 and Gaussian light-sheet with fixed HT22 cells double stained with Alexa488 anti-tubulin (Green) and Alexa560 anti-Tom20 (mitochondria outer membrane, Red). The sample is scanned by LBS1, LBS2 and Gaussian light-sheets, as shown in (a), (b) and (c) respectively. All scanning is done with 100 nm step size. Deconvolution is applied to obtain (b) in order to remove the special structure in the PSF. In each image, upper panel shows the lateral projection (towards the coverslip), and the lower panel shows the axial cross section along the dotted blue line in the upper panel. Gaussian light-sheet (600nm FWHM thickness, positioned for best resolution at the cell bottom on the coverslip) generates obviously more background than LBS at positions above the cell bottom. (d) The measured optical transfer function (OTF) of the imaged microtubule (green channel), which confirms that LBS2 has the best axial resolution (2.5 μm^{-1} or 400 nm), and LBS1 has better axial resolution (1.75 μm^{-1} or 570 nm) and much lower background than Gaussian light-sheets (1.4 μm^{-1} or 700 nm). Scale bars: 5 μm.

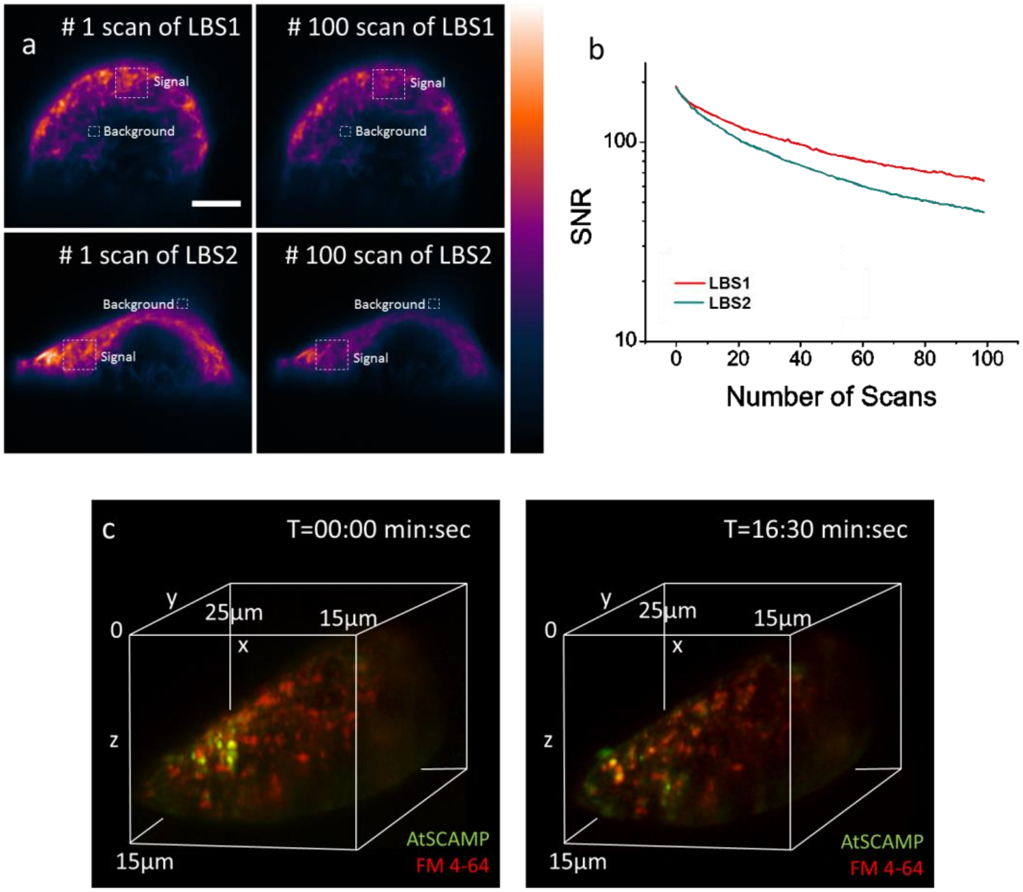

Supplementary Figure 4 | Photobleaching of LBSs. Microtubules in HT22 cells are continuously scanned by LBS over a range of 40 μm using 200 nm step size and 30 ms exposure time at each layer. The total laser power reaching the sample for LBS1 and LBS2 was 24.8 μW and 26.6 μW so that the peak intensity is the same for both LBSs. The cells with similar size, morphology and brightness were selected for imaging. The mean signal to noise ratio at any given time point was calculated according to a specified region from the slice of image at the middle of a cell. (a) The slices used to calculate SNR from the first scan (left) and the 100th scan (right) for LBS1 (upper) and LBS2 (lower). SNR for each time point is calculated based on the mean value of the boxed region (marked as 'signal') to the S.D. of the background region (marked as 'background'). Figures are displayed in the same dynamic range with "Gem" lookup table. (b) The plot of descending SNR for LBS1 and LBS2 over 100 times of 3D scans. LBS1 with its more energy localized to the central peak, exhibit a lower rate of bleaching compared to LBS2 where more power is required to have the same SNR. Both LBSs shows a low rate of bleaching in general. (c) The first time point (left) and the 16 min 30 s (the 1000th time point) of a live plant root BY-2 cells imaged by LBS1 using 20 μW laser power at 100 frame per second, showing the small SNR reduction after long term imaging.

Supplementary Video 1 | LBS generation at different wavelengths with a fixed set of slit and annulus. Simulation confirms that the same LBS pattern can be produced at any wavelength from 405nm to 1100nm using a fixed set of single slit and annulus.

Supplementary Video 2 | 4D multicolor imaging of endocytosis Tobacco BY-2 cells. Green: AtSCAMP3-GFP; Red: FM 4-64. Video is taken by LBS1 and recorded at a rate of 100Hz per frame per color. Each volume stack contains 50 two-color slices yielding a time resolution of 1s. Scale bars: 5μm.
